# A prospective study of peer victimization and depressive symptoms among left-behind children in rural China: the mediating effect of stressful life events

**DOI:** 10.1186/s13034-022-00485-8

**Published:** 2022-06-29

**Authors:** Xiaoqing Zhang, Houlin Hong, Wei Hou, Xia Liu

**Affiliations:** 1 School of Health Professions, Stony Brook University, New York, USA; 2 School of Public Health and Health Policy, City University of New York, New York, USA; 3 Department of Family, Population, and Preventive Medicine, Stony Brook University, New York, USA; 4 Institute of Developmental Psychology, Faculty of Psychology, Beijing Normal University, Beijing, China; 5 No. 19 Xinjiekouwai Street, Beijing, 100875 China

**Keywords:** Depressive symptoms, Left-behind children, Peer victimization, Stressful life events

## Abstract

**Background:**

The Ministry of Civil Affairs of the People’s Republic of China reported that in 2018, 6.97 million left-behind children (LBC), children who live in rural areas away from their parents, were being cared for by grandparents, relatives, elder siblings, or often living alone. Their parents have migrated to cities for better income opportunities. While a number of studies have detailed elevated depressive symptoms among LBC, relatively little is known about the causes of poorer mental health in LBC.

**Methods:**

This study used longitudinal data to examine associations between peer victimization, stressful life events, and depressive symptom severity in LBC from four randomly-selected middle schools in Guizhou Province, China. A total of 862 children aged 11–18 years, with 472 LBC (54.76%) and 390 non-left-behind children (NLBC) (45.24%), were included in the analysis. T-test was used to compare the depressive symptoms, peer victimization, and stressful life events between LBC and NLBC at baseline and follow-up (6 months later). Multivariable linear regression models were used to examine the mediation effect of follow-up stressful life events on the relationship between baseline peer victimization and follow-up depressive symptoms among LBC.

**Results:**

Results suggested that LBC had higher peer victimization and stressful life events than NLBC (β = 1.28, p = 0.04), and peer victimization and stressful life events at baseline were associated with increased follow-up depressive symptoms among LBC (Peer victimization: β = 0.25, p < 0.0001; Stressful life events: β = 0.15, p < 0.001). Peer victimization affected depressive symptoms partially through stressful life events for female LBC and completely through stressful life events for male LBC, controlling for age, perceived socioeconomic status, and school.

**Conclusions:**

This study highlights the vulnerability of LBC exhibiting negative mental health outcomes as they were found to experience more peer victimization and feel more stressed when stressful life events happened, compared with NLBC. Results of this study suggested that protecting LBC from peer victimization experiences can potentially prevent LBC from experiencing an increased impact of other stressful life events, thus decreasing the likelihood of their depressive symptoms. Intervention design should consider the different mediating effects of stressful life events on the relationship between peer victimization and depressive symptoms among female and male LBC.

## Background

Since the late 1970s, China has experienced rapid industrialization alongside the creation of a capitalism-based market to replace the prior communism-based system. During this time of rapid industrialization, there was an intense population shift as millions of low-income farmers were motivated to move to urban areas for higher income opportunities. Some migrating workers left behind not only their social capital and network but also their children. The Hukou (household registration) policy in China prevents migrants and their children from urban residency rights and many of the social benefits associated with those rights, including public housing, medical care and social insurance, resulting in parents’ having to expend more effort and money if they want to move to cities with their children. In 2018, the Ministry of Civil Affairs of the People’s Republic of China announced that there were 6.97 million of these so-called “left-behind children” (LBC) being cared for by a remaining parent, grandparents, relatives, or elder siblings, or living alone in rural areas [1]. The sheer magnitude of this phenomenon makes it a societal problem that deserves scientific attention, especially when there is numerous research suggesting that LBC are at a greater risk of exhibiting negative mental health outcomes.

Depression is an episodic, recurring disorder characterized by persistent and pervasive sadness or unhappiness and associated symptoms such as negative thinking, lack of energy, difficulty concentrating, loss of appetite, and sleep disturbances [2]. Childhood depression impacts healthy growth and development, school performance, psychosocial functioning, and the development of other psychiatric disorders in later life [2]. Compared with children living with both parents, LBC have consistently been found to have higher depressive symptoms [2–4]. For example, the prevalence of depressive symptoms was 33.7% in the LBC group and 22.8% in the NLBC group, based on the result of a meta-analysis of 30 comparative studies consisting of 22,148 LBC and 20,049 NLBC [5]. Because the large number of LBC and their elevated risk of experiencing depressive symptoms have posed a major threat to LBC’s long-term mental and physical wellbeing as well as to public health, it is vital to study LBC’s depressive symptoms and associated factors to improve our understanding of the characteristics and mechanism of depressive symptoms in LBC, which will have important implications for designing intervention programs.

### Peer victimization and depressive symptoms among LBC

As LBC do not have their parents living with them for close supervision and care, they are more vulnerable to peer victimization [6, 7]. As one form of dysfunctional peer relationships, peer victimization is defined as experiencing maltreatment from peers, including physical, verbal, and relational aggression. Chen et al. [6] conducted a study in rural China on one representative sample of 800 middle school students from grades seven to nine and reported that children left behind by their fathers experienced more peer victimization than their counterparts who live with both parents [6]. In addition, Zhang et al. [7] surveyed 742 LBC in grades six to ten from rural China. The results indicated that a total of 31.6% of the LBC reported recurrent bullying victimization, and the number was significantly higher than that of children living with both parents (N = 1405; 25.2% reported bullying victimization) [7].

Theoretical and empirical perspectives suggest a link between peer victimization and depressive symptoms [8–10]. For example, Gilbert [11] illustrated how attacks on peer-group rank, which has strong similarities to physical victimization, contribute to depression [11]. In addition, Interpersonal Risk Model posits that poor peer relationships are a significant stressor contributing to problematic outcomes, such as depressive symptoms [8], and peer victimization is a manifestation of poor peer relationships. Empirical research has also shown correlations between peer victimization and depressive symptoms. For example, Hawker & Boulton [10] conducted a meta-analysis and concluded that peer victimization was most strongly related to depression, less so to loneliness and self-esteem, and least to anxiety [10]. More recently, Zhao et al. [12] surveyed 478 first and second-year middle school students to explore the features of peer victimization and the relationship between peer victimization and depression among early Chinese adolescents [12]. Their results showed that early adolescents exhibited a high prevalence of peer victimization, and peer victimization was positively correlated with depression among those children. However, existing research has focused on the impact of peer victimization on LBC’s depressive symptoms at the same point in time, but has not explored the long-term effects of peer victimization. Does peer victimization experienced early on have a significant impact on LBC’s subsequent depressive symptoms over time? Exploring the longitudinal effects will help us further understand the relationship between peer victimization and adolescent depressive symptoms, which will provide useful guidance for designing intervention programs to prevent LBC from depressive symptoms.

### The mediating effect of stressful life events

Much of the research about LBC has focused directly on the child’s exposure and reaction to stressful life events [13–16], in essence viewing the participants as double-victims of being left behind and of other stressful life events such as interpersonal conflict, academic stress, corporal punishment, and physical health problems. While likely to be true, this view does not recognize the role of other individuals in actively stigmatizing LBC. LBC have reported experiencing more peer victimization when other children bully them willfully, knowing that they are children left behind by their parents. Identifying mechanisms linking peer victimization and stressful life events to LBC’s depressive symptoms is vital to designing effective prevention interventions targeting those processes.

Peer victimization may contribute to depressive symptoms in LBC through stressful life events. According to stress sensitization theory, exposure to extreme stressors may enhance an individual’s reactivity to subsequent stressors [17, 18]. Sensitization refers to the circumstance where an organism responds more strongly to a variety of previously neutral stimuli after exposure to a potentially threatening stimulus. Smid et al. [19] validated the stress sensitization hypothesis with their research findings that residents whose house was completely destroyed by the disaster (a major explosion of a fireworks depot resulting in 22 deaths and about 1000 injured residents) responded more strongly to stressful life events 18–20 months after the disaster than residents who reported less extreme disaster exposure [19]. As peer victimization is a potent type of stressor for adolescence [20], it is likely that peer victimization may engender heightened reactivity to subsequent stressful life events. In other words, peer victimization can be associated with a perceived increase in stressful life events.

On the other hand, stressful life events have been consistently reported to be correlated with an increase in depressive symptoms and the onset of major depression in adolescents [21, 22]. Relevant literature also shows that stressful life events are linked to increased depressive symptoms [13, 23]. It is possible that stressful life events play a mediating role in the relationship between peer victimization and depressive symptoms. In fact, stressful life events have been reported to be a mediator in emotional adaptation studies. For example, assaultive violence and a family history of mood disorder contribute to depression through stressful life events [24, 25]. Based on the stress sensitization theory and the empirical research results suggesting the mediator role of stressful life events, we hypothesize that stressful life events mediate the positive correlation between peer victimization and depressive symptoms in LBC.

While both peer victimization and stressful life events were found to be positively associated with depressive symptoms, no empirical studies have examined the linkage between peer victimization, stressful life events, and depressive symptoms among LBC. Because LBC are more likely to encounter peer victimization or be the target of aggression by peers, it is possible that peer victimization is a more important factor as it predicts a higher impact of other stressful life events, thereby contributing to more depressive symptoms. A better understanding of the relationship mechanisms between peer victimization, stressful life events, and depressive symptoms can inform effective intervention programs that target the most important factor to improve LBC’s mental health.

### The present study

Using data from a prospective survey of 1,134 children from four randomly selected middle schools located in the Guizhou province in China, the primary aim of this study is to explore the longitudinal effects of peer victimization in addition to other stressful life events on the depressive symptoms of LBC, and to further examine the mechanism of how peer victimization contributes to depressive symptoms in LBC, which might occur through heightened reactions to stressful life events. First, we examined the differences in peer victimization and stressful life events between LBC and NLBC. Second, we hypothesized that peer victimization and stressful life events at baseline would be positively correlated with follow-up depressive symptoms among LBC 6 months later, controlling for gender, age, perceived socioeconomic status (SES), and school. Furthermore, we hypothesized that stressful life events mediate the relationship between peer victimization and the depressive symptoms of LBC. Considering that gender differences in peer victimization and depression have been well documented; in particular, males generally report greater victimization than females, and being female has been found to be associated with more depressive symptoms among LBC [4, 26], the mediation model testing was repeated by gender to examine whether the model applies equally to both males and females.

## Methods

### Study sample

The longitudinal data collected involves 1134 children, left-behind and non-left-behind, male and female, aged between 11 and 18, from four randomly selected middle schools in Guizhou province, one of the areas with the highest LBC concentration [1]. With the help of teachers and school administrators, consent and assent forms were sent to the participants and caregivers prior to the study. The research team distributed questionnaires in the classrooms to participants who completed both the consent and assent process. The research staff monitored survey completion and answered questions. The survey lasted approximately 45 min. A total of 1134 students completed the study at baseline in December 2017, and at follow-up 6 months later in June 2018. Participants received T-shirts as a reward for their participation. The study was approved by the Human Research Ethics Committee of Beijing Normal University, and the current research using the de-identified dataset was approved by the Institutional Review Board of Stony Brook University.

### Variables and procedure

#### Left-behind children (LBC)

In the current study, children with one or both parents having migrated are considered LBC coded as 1; children living with both parents are considered NLBC coded as 0.

#### Depressive symptoms

The outcome variable is depressive symptoms, measured by the Center for Epidemiological Studies Depression Scale for Children (CES-DC) [27]. CES-DC is a 20-item 4-point self-report Likert scale (0 = not at all, 1 = a little, 2 = some, and 3 = a lot). Respondents were asked to describe how often they experienced the symptoms stated in each item during the past week. Sample items include “I was bothered by things that usually don’t bother me,” “I felt like I was too tired to do things,” “I felt lonely like I don’t have any friends,” and “It was hard to get started doing things.” We calculated the total score to measure each respondent’s depressive symptoms. A higher score indicates that the individual experiences depressive symptoms more often. CES-DC has been reported to have good reliability and validity among Chinese children and adolescents [26, 28]. Cronbach’s alphas of depressive symptoms for the current study are 0.80 and 0.81 at baseline and follow-up.

#### Peer victimization

An adapted version of the Multidimensional Peer-Victimization Scale (MPVS) [29] was used to assess peer victimization. The scale consists of 21 items, all of which is a 4-point Likert scale with 0 translating to “never happen,” 1 to “seldom happened,” 2 to “happened sometimes,” and 3 to “always happened.” Sample questions include: “During the semester, other classmates have pushed or kicked me (physical victimization);” “During the semester, other classmates have cursed me (verbal victimization);” “During the semester, other classmates have asked my friends not to play with me or talk to me (social manipulation);” “During the semester, other classmates have stolen my money or belongings (attacks on property).” We used the total score, and a higher score means more peer victimization experiences. MPVS has been used among Chinese children and has shown good reliability and validity [30]. Cronbach’s alphas of peer victimization for the current study are 0.92 and 0.92 at baseline and follow-up.

#### Stressful life events

Stressful life events were measured with the Adolescent Self-Rating Life Events Checklist [31], which included 27 items falling into six dimensions: interpersonal conflict (e.g., conflicts with classmates or friends), academic stress (e.g., failures in the exam; heavy course load), punishment (e.g., criticism or physical punishment); loss (e.g., death of a loved one or loss of property); health and adaptation problems (e.g., severe illnesses and maladjustment to changed diet, daily routine or living environments) and all other types of events. The checklist is a six-point Likert scale (1 = “negative events never happened,” 2 = “no impact,” and 6 = “extremely high impact”). We used the total score, and a higher score indicates that the participant experiences a higher level of stress. ASLEC has good reliability and validity in the Chinese population [31]. Cronbach’s alphas of stressful life events for the current study are 0.89 and 0.90 at baseline and follow-up.

Previous research has documented that socio-demographic variables such as age, gender, and perceived SES [4, 32], were associated with depressive symptoms among LBC. Thus, age, gender, and perceived SES were included as control variables. In addition, as students were from four different schools, school was also included as a covariate to control for potential effects. Perceived SES was measured with a Likert scale from 01 being very low socioeconomic status to 10 being very high [33]. Observations with more than 20 percent of the questions missing in depressive symptoms, peer victimization, and stressful life events were excluded from the analysis.

### Statistical analysis

Demographic variables were compared between LBC and NLBC using the Mann–Whitney test for continuous measures and the Chi-square test for categorical measures. T-test was used to compare depressive symptoms, peer victimization, and stressful life events at baseline and follow-up. Furthermore, linear mixed-effect modeling was used to examine whether there was a significant difference in depressive symptoms, peer victimization, and stressful life events between LBC and NLBC at baseline and follow-up after adjustment for age, gender, perceived SES, and school. The interactions between time and LBC were also tested for significance, and the insignificant interactions were removed from the final model. To evaluate whether baseline peer victimizations and baseline stressful life events were positively correlated with follow-up depressive symptoms among LBC, multiple regression was used with adjustment for age, gender, perceived SES, and school. For LBC, the mediating effect of follow-up stressful life events on baseline peer victimization predicting follow-up depressive symptoms was examined using four regression models as follows: (1) baseline peer victimization predicting follow-up stressful life events; (2) follow-up stressful life event predicting follow-up depressive symptoms; (3) baseline peer victimization predicting follow-up depressive symptoms; (4) baseline peer victimization and follow-up stressful life events predicting follow-up depressive symptoms. All four models included gender, age, perceived SES, and school as covariates. The same analyses were repeated for female and male LBC separately. All statistical analyses were performed using SAS 9.4.

## Results

### Demographic information

A total of 1134 middle school students completed the survey at both baseline and follow-up. However, 272 participants were excluded from the analysis due to a change in left-behind status, either from LBC to NLBC or vice versa. As a result, 862 children were included in the final analysis. The median age of the total sample was 14 years (interquartile range (*IQR*) 13, 14)), with 405 male students and 457 female students. Of the 862 study participants, 390 were NLBC, of which 196 (50.26%) were males; and 472 were LBC, of which 209 (44.28%) were males. The median age for the NLBC and LBC was 13 (*IQR* 13, 14) and 14 (*IQR* 13, 14) years, respectively (Table 1).

**Table 1:** Demographics

Variables	Overall (N = 862)	NLBC (N = 390)	LBC (N = 472)	
	Median (IQR)	Median (IQR)	Median (IQR)	P value
Age	14 (13, 14)	13 (13, 14)	14 (13, 14)	0.04
Perceived SES	4 (3, 5)	4 (3, 5)	4 (3, 5)	0.28
	N (%)	N (%)	N (%)	
Male	405 (46.98%)	196 (50.26%)	209 (44.28%)	

*LBC* left-behind children, *NLBC* non-left-behind children, *IQR* interquartile range, *SES* perceived socioeconomic status

### Outcome comparisons between LBC and NLBC

T-test was used to compare the depressive symptoms, peer victimization, and stressful life events at baseline and follow-up. The LBC group showed a higher score in all three dimensions at baseline and follow-up (Table 2).

**Table 2:** Comparison of depressive symptoms, peer victimization, and stressful life events at baseline and follow-up

Variable	NLBC (N = 390)	LBC (N = 472)	
	Mean (SD)	Mean (SD)	P value
Depressive symptoms			
Baseline	19.17 (9.67)	20.55 (9.31)	0.03
Follow-up	20.29 (10.36)	21.21 (10.59)	0.2
Peer victimization			
Baseline	32.70 (9.28)	34.19 (9.74)	0.02
Follow-up	33.37 (9.30)	35.60 (10.60)	0.001
Stressful life events			
Baseline	55.68 (17.44)	58.91 (16.36)	0.005
Follow-up	55.26 (17.55)	58.17 (17.11)	0.01

*LBC* left-behind children, *NLBC* non-left-behind children, *SD* standard deviation

Time, gender, and perceived SES were significantly associated with depressive symptoms. There was a 0.98 unit increase of CES-DC score over time (t (843) = 2.7, p = 0.007). Male students showed −2.78 (t (856) = −4.63) units less CES-DC score compared to female students. Perceived SES was negatively associated with CES-DC score (β = −0.55, t (843) = −3.51, p = 0.0005). For peer victimization, time was significant that at follow-up, students had a 1.52 unit higher peer victimization (t (845) = 4.15, p < 0.0001). LBC reported a 1.28 unit higher peer victimization than NLBC (t (856) = 2.08, p = 0.04). Perceived SES showed a protective effect that every unit increase in perceived SES was associated with a 0.72 decrease in peer victimization (t (845) = −4.66, p < 0.0001). Students from school 2 showed significantly less peer victimization compared to school 4, with 3.12 units less (p < 0.0001). LBC status and perceived SES were significantly associated with stressful life events. LBC had 2.49 units higher stressful life events than NLBC (t (855) = 2.28, p = 0.02). A unit increase in perceived SES showed a −0.92 (t (844) = −3.46, p = 0.0006) unit decrease in stressful life events (Table 3).

**Table 3:** Regression coefficients of depressive symptoms, peer victimization, and stressful life events

Variables		Estimate	Standard Error	T value	P value
Depressive symptoms					
Intercept		21.96	3.58	6.14	< .0001
Time	Follow-up vs. Baseline	0.98	0.36	2.7	0.007
LBC	LBC vs. NLBC	0.83	0.63	1.32	0.19
Age		0.07	0.26	0.27	0.79
Gender	Male vs. Female	−2.78	0.60	−4.63	< .0001
School	1 vs. 4	−0.14	1.10	−0.13	0.90
School	2 vs. 4	−0.32	0.79	−0.4	0.69
School	3 vs. 4	1.20	0.86	1.4	0.16
SES		−0.55	0.16	−3.51	0.0005
Peer Victimization					
Intercept		41.80	3.50	11.93	< .0001
Time	Follow-up vs. Baseline	1.52	0.37	4.15	< .0001
LBC	LBC vs. NLBC	1.28	0.61	2.08	0.04
Age		−0.34	0.25	−1.35	0.18
Gender	Male vs. Female	0.69	0.58	1.19	0.23
School	1 vs. 4	−1.41	1.06	−1.32	0.19
School	2 vs. 4	−3.12	0.77	−4.06	< .0001
School	3 vs. 4	−0.84	0.84	−1	0.32
SES		−0.72	0.15	−4.66	< .0001
Stressful life events					
Intercept		59.80	6.18	9.67	< .0001
Time	Follow-up vs. baseline	−0.45	0.63	−0.72	0.47
LBC	LBC vs. NLBC	2.49	1.09	2.28	0.02
Age		0.11	0.45	0.25	0.81
Gender	Male vs. Female	−1.42	1.04	−1.36	0.17
School	1 vs. 4	2.08	1.90	1.09	0.27
School	2 vs. 4	−2.67	1.37	−1.94	0.052
School	3 vs. 4	0.88	1.49	0.59	0.56
SES		−0.92	0.26	−3.46	0.0006

*LBC* left-behind children, *NLBC* non-left-behind children, *SES* perceived socioeconomic status

### Correlations between peer victimization, stressful life events, and depressive symptoms among LBC

We further examined whether baseline peer victimization and stressful life events were positively correlated with follow-up depressive symptoms among LBC. Male LBC showed less depressive symptoms than female LBC (β = −3.85, t (466) = 4.6, p < 0.0001). Peer victimization at baseline significantly predicted follow-up depressive symptoms (β = 0.25, t (466) = 4.6, p < 0.0001). Stressful life events at baseline were also significantly associated with follow-up depressive symptoms (β = 0.15, t (466) = 4.6, p < 0.0001) (Table 4).

**Table 4:** Regression coefficients examining follow-up depressive symptoms among left-behind children

Variable		Estimate	Standard error	T value	P value
Intercept		5.62	6.69	0.84	0.4011
Peer victimization baseline		0.25	0.059	4.6	< .0001
Stressful life events baseline		0.15	0.03	4.53	< .0001
Gender	Male vs. Female	−3.85	0.89	−4.32	< .0001
Age		0.45	0.43	1.03	0.3048
Perceived SES		−1.17	0.32	−3.63	0.0003
School 1	1 vs. 4	−0.75	1.75	−0.43	0.6699
School 2	2 vs. 4	−1.13	1.14	−0.99	0.3229
School 3	3 vs. 4	−1.13	1.13	−1	0.3184

*SES* perceived socioeconomic status

**Fig. 1:**
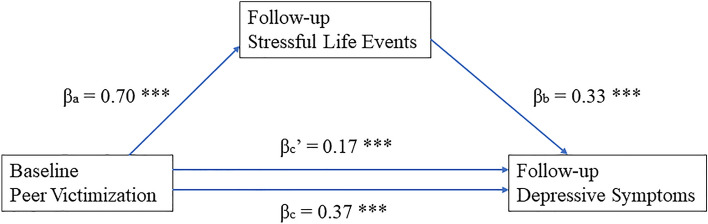
Stressful life events mediate the relationship between peer victimization and depressive symptoms. *p < 0.05, **p < 0.01, ***p < 0.001. c indicates the direct relationship between baseline peer victimization and follow-up depressive symptoms; c’ indicates the same relationship after controlling for follow-up stressful life events

As Fig. 1 shows, the relationship between baseline peer victimization and follow-up depressive symptoms was partially mediated by follow-up stressful life events. First, follow-up stressful life events were correlated both with baseline peer victimization and follow-up depressive symptoms (p values for both regression coefficients < 0.001). In addition, baseline peer victimization independently significantly affected follow-up stressful life events and follow-up depressive symptoms (p values for both regression coefficients < 0.0001). When follow-up stressful life events and follow-up depressive symptoms were simultaneously included in the same model, the regression coefficient of baseline peer victimization on follow-up depressive symptoms dropped from independent regression coefficient 0.37 to joint 0.17, although remaining significant. This result indicates a partial mediation effect of follow-up stressful life events. The significance of this partial mediation was validated using the Bootstrap method.

**Fig. 2:**
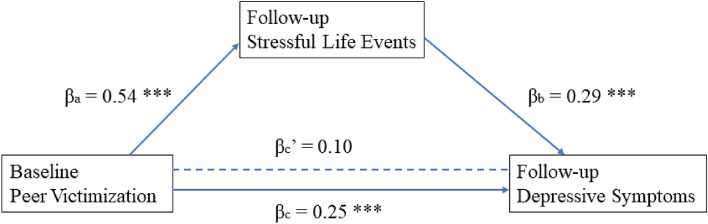
Stressful life events mediate the relationship between peer victimization and depressive symptoms among male LBC. *p < 0.05, **p < 0.01, ***p < 0.001. c indicates the direct relationship between baseline peer victimization and follow-up depressive symptoms; c’ indicates the same relationship after controlling for follow-up stressful life events

**Fig. 3:**
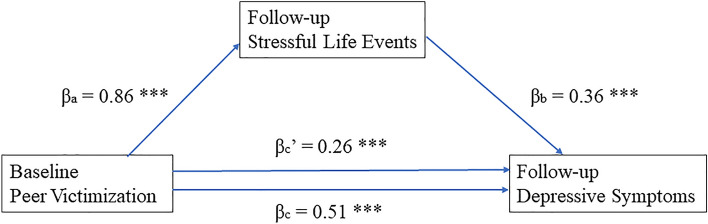
Stressful life events mediate the relationship between peer victimization and depressive symptoms among female LBC. *p < 0.05, **p < 0.01, ***p < 0.001. c indicates the direct relationship between baseline peer victimization and follow-up depressive symptoms; c’ indicates the same relationship after controlling for follow-up stressful life events

The mediating effects were first examined among male LBC, then among female LBC separately. The results were as follows: For male LBC, when baseline peer victimization and follow-up stressful life events were included in the same model, baseline peer victimization became an insignificant predictor, suggesting follow-up stressful life events had a full mediating effect on the relationship between baseline peer victimization and follow-up depressive symptoms (Fig. 2); for female LBC, when baseline peer victimization and follow-up stressful life events were included in the same model, both of the predictors remained significant, but their regression coefficients decreased, thus showing that follow-up stressful life events partially mediated the relationship between baseline peer victimization and follow-up depressive symptoms among female LBC (Fig. 3).

## Discussion

The millions of LBC who live in rural China and their high depression risk have made the LBC phenomenon a public health concern and societal problem. In an attempt to alleviate the mental health burden borne by those children, it is vital to identify the most relevant risk factors and understand the mechanisms among risk factors linked to LBC’s poorer mental health outcomes. The current study provides theoretical guidance for the mechanism of depressive symptoms in LBC, and serves as a reference for depression prevention of LBC in other countries such as Indonesia, Thailand, and Sri Lanka [34, 35]. For example, LBC in Indonesia and Thailand were reported to be more likely to have poor psychological outcomes compared to NLBC [34]. The results of the current study may offer theoretical guidance for these countries.

The results of this study suggest that LBC encounter higher peer victimization than NLBC, and peer victimization is associated with an increase in LBC’s follow-up depressive symptoms. Peer victimization among adolescents is a pressing issue, especially among LBC. Compared to children living with both parents, children living with a single parent or step/cohabiting family have significantly elevated rates of victimization, including maltreatment, physical assault, exposure to community violence, and sexual victimization [36]. LBC’s parents are not physically present with them in their hometown, and LBC’s guardians are mostly grandparents. Lack of close parental monitoring and care could contribute to LBC’s vulnerability to peer victimization. A government report [37] on adolescents (A Study on Six Unique Adolescent Groups Requiring Special Government Attention in China) shows that 47.6% of 4,533 LBC indicated being bullied, including being verbally abused (63.9%), looked down upon (36.3%), and beaten up (24.2%) [37]. As the results of this study show that peer victimization is related to LBC’s increased depressive symptoms, it is vital for the government, schools, and communities to take measures to protect LBC from peer victimization. For example, schools can implement bullying prevention programs by offering student lessons that involve short films about bullying, discussion, group work, and role-play exercises, so that LBC have an increased awareness of peer victimization and learn how to seek help in a timely manner.

More importantly, peer victimization was found to affect LBC’s depressive symptoms through stressful life events. Peer victimization negatively affects LBC’s emotional adaptation to stressful life events, leaving them more susceptible to the negative feelings induced by common stressful life events that happen to them. As a consequence, those children reported a higher level of stress at the follow-up study, which was linked to their increased follow-up depressive symptoms. Because of the negative emotions elicited by peer victimization experiences, LBC exhibit high levels of emotional arousal and reactivity [38]. Gradually, their abilities to manage their emotions diminish when they face stressful life events, making them more vulnerable to depressive symptoms. This study provided empirical results suggesting that stressful life events are a mediator of the association between peer victimization and depressive symptoms of LBC.

Furthermore, peer victimization was found to affect depressive symptoms partially through stressful life events for female LBC and completely through stressful life events for male LBC. For female LBC, peer victimization not only affects depressive symptoms through stressful life events, but also directly affects depressive symptoms. According to the response style theory [39], females are more prone to rumination when adverse events happen, whereas males turn to distraction or problem-solving. Research results have suggested that when people ruminate in the context of a dysphoric mood, they recollect more negative memories from the past, interpret their current situation in a more negative way, and feel more pessimistic about their future [40, 41]. Thus, rumination was found to predict the severity of depressive symptoms [42]. After experiencing peer victimization, female LBC’s rumination tendency may prolong and enhance the negative thinking associated with depressed mood, which in turn may lead to more depressive symptoms over time. In contrast, when male LBC are distracted from their ruminations or attempt to problem-solve, they may not experience a depressed mood due to peer victimization. However, peer victimization does reduce male LBC’s abilities to cope with stressful life events. As a result, male LBC reported a high level of stress in the follow-up study, which was associated with their increased follow-up depressive symptoms. The gender difference in mediating effects can also be related to gender role expectations [43]. For example, men are expected to be strong and fight back, which may prevent men from depressive symptoms. Instead, they are more inclined to exhibit externalizing behaviors such as aggression [44]. However, the perspective of gender role expectations needs to be further explored. In terms of designing intervention programs to prevent LBC from peer victimization, several existing school-based programs can serve as references. For instance, the New National Initiative against Bullying comprised more than 100 schools with approximately 21,000 students from grades 4–7 in Norway [45]. Students were administered the Bully/Victim Questionnaire before and after 8 months of intervention with the Olweus Bullying Prevention Program. Results suggested substantial reductions (by 32–49%) in bully/victim problems. Future research should examine how to adapt available programs in the context of China. Relevant literature from China offers general suggestions such as using a holistic approach to involve all stakeholders, including LBC, their parents, school, and teacher, as well as strengthening LBC’s peer relationship-building skills and educating parents on caring for their children’s emotional needs [7]. Research focusing on protective factors that can prevent LBC from exhibiting adverse mental outcomes will have important implications on effective intervention program design. Finally, female and male LBC could be offered separate programs because peer victimization was found to affect their depressive symptoms differently.

Some limitations of this study should be noted. First, participants in this study were from one town in the Guizhou Province. Thus, the generalizability of the study findings to LBC in other areas of China is limited. Second, the present research looked at LBC who are victims of bullying. However, some of them might also be involved in bullying others [46]. Future studies can explore whether there is any difference in their mental health outcomes between pure victims and bully-victims for LBC. Third, we only conducted one follow-up study 6 months later, which limits the understanding of how long the longitudinal effects will last. Future research can carry out multiple follow-up studies to increase the credibility of the conclusion. Lastly, it is also worth mentioning that we excluded children from the sample whose left-behind status changed during the course of the study, because their status change could potentially affect their victimization experiences and stressful life events, as well as their depressive symptoms. Future research can examine what these effects are for children whose status changed from LBC to NLBC and for those whose status changed from NLBC to LBC, to demonstrate the impact of parental absence.

## Conclusions

To conclude, this is the first study investigating the longitudinal effects of peer victimization and stressful life events on Chinese LBC’s depressive symptoms, as well as the mediating effect of follow-up stressful life events on the relationship between baseline peer victimization and follow-up depressive symptoms of LBC, adjusting for gender, age, perceived SES, and school. This study highlights the vulnerability of LBC exhibiting negative mental health outcomes as they were found to experience more peer victimization and feel more stressed when stressful life events happened, compared with NLBC. Still more troubling, both peer victimization and stressful life events at baseline were found to be positively correlated with the follow-up depressive symptoms of LBC. More importantly, as peer victimization experiences are developmentally salient stressors among adolescents and are related to the development of their psychopathology, it is particularly vital to understand the mechanisms linking peer victimization to adolescents’ adverse mental health outcomes. Results of this study revealed that follow-up stressful life events were a mediator of the association between baseline peer victimization and changes in follow-up depressive symptoms among LBC. Future research can focus on designing effective prevention programs for LBC.

## Data Availability

The datasets used and/or analysed during the current study are available from the corresponding author on reasonable request.

## Abbreviations

CES-DC: Center for Epidemiological Studies Depression Scale for Children
IQR: Interquartile Range
LBC: Left-behind children
MPVS: Multidimensional Peer Victimization Scale
NLBC: Non-left-behind children
SES: Socioeconomic status
